# Wildfire smoke impacts respiratory health more than fine particles from other sources: observational evidence from Southern California

**DOI:** 10.1038/s41467-021-21708-0

**Published:** 2021-03-05

**Authors:** Rosana Aguilera, Thomas Corringham, Alexander Gershunov, Tarik Benmarhnia

**Affiliations:** 1 Scripps Institution of Oceanography, University of California San Diego, La Jolla, CA USA; 2 Herbert Wertheim School of Public Health and Human Longevity Science, University of California San Diego, La Jolla, CA USA

**Keywords:** Climate sciences, Epidemiology

## Abstract

Wildfires are becoming more frequent and destructive in a changing climate. Fine particulate matter, PM_2.5_, in wildfire smoke adversely impacts human health. Recent toxicological studies suggest that wildfire particulate matter may be more toxic than equal doses of ambient PM_2.5_. Air quality regulations however assume that the toxicity of PM_2.5_ does not vary across different sources of emission. Assessing whether PM_2.5_ from wildfires is more or less harmful than PM_2.5_ from other sources is a pressing public health concern. Here, we isolate the wildfire-specific PM_2.5_ using a series of statistical approaches and exposure definitions. We found increases in respiratory hospitalizations ranging from 1.3 to up to 10% with a 10 μg m^−3^ increase in wildfire-specific PM_2.5_, compared to 0.67 to 1.3% associated with non-wildfire PM_2.5_. Our conclusions point to the need for air quality policies to consider the variability in PM_2.5_ impacts on human health according to the sources of emission.

## Abstract (web summary)

Recent toxicological studies suggest that wildfire particulate matter may be more toxic than equal doses of ambient PM_2.5_. Here, the authors show that even for similar exposure levels, PM_2.5_ from wildfires is considerably more dangerous for respiratory health at the population level.

## Introduction

Fine particulate matter, i.e., particles with aerodynamic diameter ≤2.5 μm (PM_2.5_), is the main component of wildfire smoke^1^ that impacts public health^2–5^. PM_2.5_ can be inhaled into the deepest recesses of the lungs^6^ and may enter the bloodstream impairing vital organs including the lungs^7^. PM_2.5_ in the United States has decreased in past decades due to environmental regulations^5,8^, with the exception of wildfire-prone areas^5^. Wildfire PM_2.5_ in the US is projected to increase with climate change along with the associated burden on human health^9^. Levels of wildfire PM_2.5_ can greatly exceed those of ambient PM_2.5_, spiking episodically within a short period of time (e.g., hours after the onset of a wildfire), and such high exposure levels may generate important health impacts. Current air quality standards specific to PM_2.5_ from the Clean Air Act Amendments do not distinguish the sources of emission or chemical composition, implicitly considering PM_2.5_ from wildfires and from other sources (e.g., ports, industrial plants, and traffic emissions) to be equally harmful to human health. This is also true in other regions of the world, as in the WHO Air Quality Guidelines (AQG)^10^ for example.

Though the differential toxicity of wildfire PM_2.5_ as compared to other ambient sources of PM_2.5_ is not well understood^11–13^, recent animal toxicological studies suggest that particulate matter from wildfires is more toxic than equal doses from other sources such as ambient pollution^14,15^. In vitro and in vivo studies have shown that mechanisms that may explain wildfire-specific PM higher toxicity include inflammation, oxidative stress^15^, or increased respiratory infection by altering pulmonary macrophages activity^16^. Wildfire particulate matter is mostly carbonaceous (with 5–20% elemental carbon and at least 50% organic carbon^17,18^) and has more oxidative potential than ambient urban particulate due to the presence of more polar organic compounds^19^. All the above compounds in wildfire smoke tend to generate more free radicals and thus have a greater potential to cause inflammation and oxidative stress in the lung than urban ambient particulate from the same region^20,21^. It is therefore imperative to differentiate between smoke and non-smoke PM_2.5_ when assessing impacts on public health.

In epidemiological studies, it has been shown that PM_2.5_ from wildfire smoke can exacerbate a range of health problems including respiratory and cardiovascular issues^4,22,23^ (although some uncertainty exists^23,24^). Yet, to date, no study has assessed the public health impact of wildfire-specific PM_2.5_ as it differs from PM_2.5_ from other sources at a fine spatial resolution (e.g., zip code) and spanning multiple wildfires over a 14-year period. Previous studies assessing wildfire smoke effects on health have often been restricted to single wildfire events due to limitations in the estimation of human exposure to wildfire-specific PM_2.5_, which typically relies on computationally demanding dynamical chemical transport models (CTMs). The most extensive study^25^ to date assessed the impacts of smoke exposure in the elderly population (≥65 years) within the Western US during a 6-year period, but was resolved at a coarser level (county) and relied in part on CTMs for quantifying wildfire-specific PM_2.5_. We compare four statistical approaches to isolate wildfire-specific PM_2.5_ from other sources. These approaches do not rely on heavy computing efforts and offer the advantage of modeling daily, zip code-level wildfire-specific PM_2.5_ over a long study period and extensive area.

In Southern California (SoCal), the dry gusty offshore (northeasterly) Santa Ana winds (SAW) start-up in the fall, peak in December, and wane in the spring^26^. SAWs are thus episodic reversals of the prevailing onshore (westerly) winds in SoCal. Early season SAWs of autumn, occurring after the long dry Mediterranean summer and before the first rains of winter, typically drive the largest wildfires, while most ignitions are human caused^27^. The Southern California traditional wildfire season thus differs from that in most other Western US regions due to its meteorological, climatic, and ignition causes^27,28^. We note, however, that recent winter and spring SAW-driven wildfires and research suggest that the SoCal wildfire season may be expanding^29^. We also note that SAWs are dormant in summer, and although summer heat-driven wildfires are becoming more prevalent lately, smoke from such fires (with the notable exception of hundreds of California wildfires burning in August 2020!) does not typically impact the coastal zone, which is the reason summer is excluded from this analysis. Dry gusty SAWs accelerate and warm on their way down coastal topography towards sea level; they not only fan and spread wildfires burning in the wildland–urban interface, but also transport smoke to densely populated coastal areas. These SAW-driven wildfires can spread faster and burn longer than fires at other times of the year^30^. Furthermore, recent work has shown that PM_2.5_ tends to increase with strong SAWs in the presence of wildfire burning upwind^31^.

In this paper, we assess the impacts on respiratory health outcomes of PM_2.5_ attributable to wildfire smoke in SoCal, as compared to PM_2.5_ from other sources. In this region, sources of non-smoke PM_2.5_, include vehicular emissions, secondary aerosols (e.g., sulfate and nitrate), soil, agricultural, and industrial emissions ^32–34^. In order to robustly isolate the health impacts of wildfire-specific PM_2.5_, we apply and compare four analytical approaches. Specifically, we implemented the following distinct methods: (i) an instrumental variable approach with a two-stage regression; (ii) a spatio-temporal multiple imputation approach, and (iii) an interaction effect approach. Lastly, we also compared these approaches to (iv) a seasonal interpolation method recently proposed by Lipner et al.^35^.

In each of these approaches, we relied on two distinct exposure variables yielding eight estimates of the differential impacts of wildfire-specific PM_2.5_. We used (i) the occurrence of strong SAWs and the presence of fire upwind and (ii) smoke plume datasets (NOAA Hazard Mapping System (HMS)) within a 160 km buffer from a wildfire perimeter to identify zip code days exposed to smoke. In addition to isolating the effect of wildfire-specific PM_2.5_ from other sources on respiratory health, the analytical methodology implemented in this study allowed us to cover a large region, population, and study period spanning 14 years (1999–2012).

**Fig. 1:**
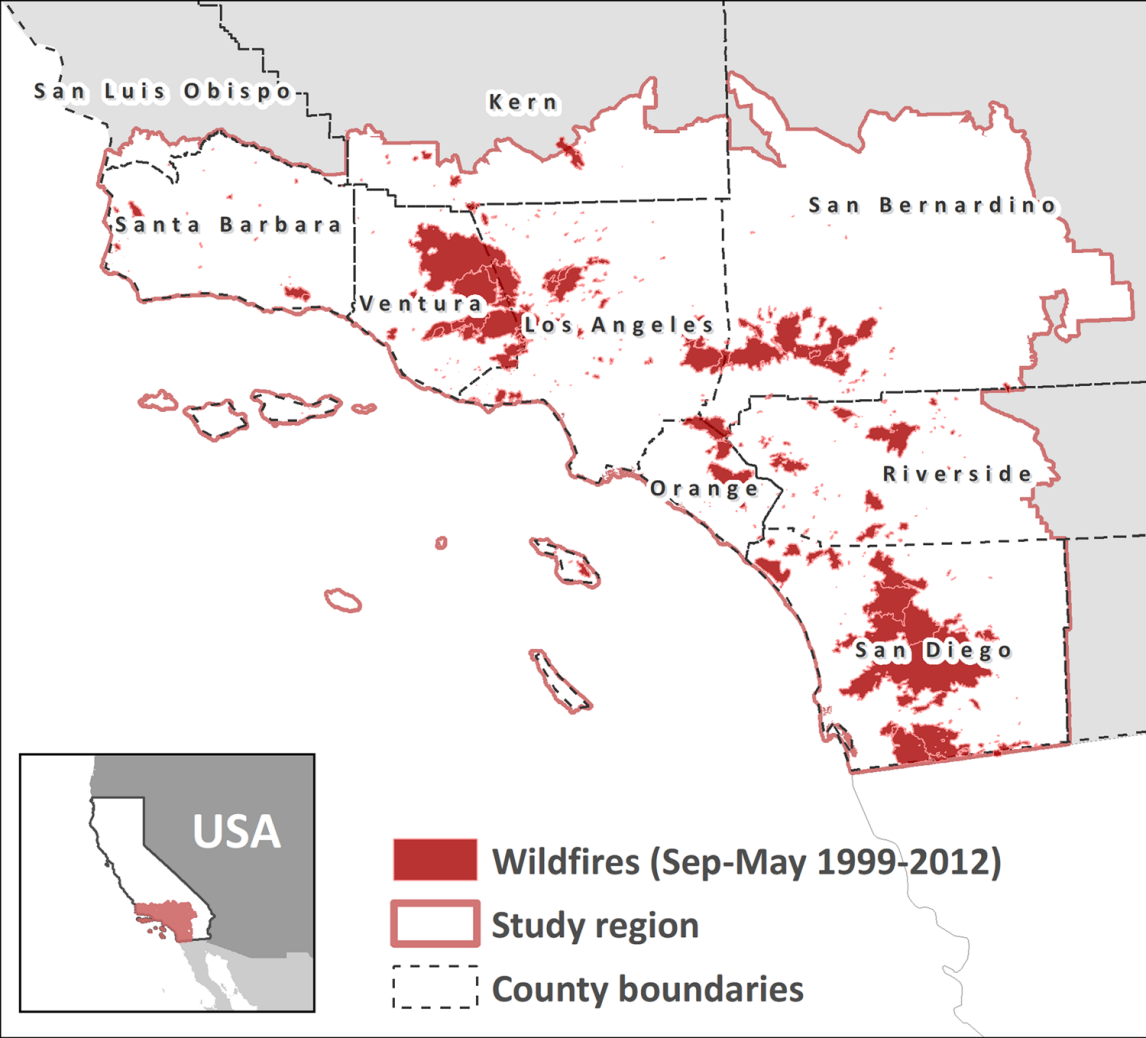
Wildfire perimeters in Southern California (1999–2012). Wildfire perimeters shown here represent the total area burned for a given fire during our study period comprising 1999–2012, excluding summer months (June, July, and August). The inset figure shows the location of our study region, which includes zip codes within the Santa Ana Wind domain in Southern California, USA.

Our study comprised 696 zip code polygons within the Santa Ana wind domain (Fig. 1). The Santa Ana wind season extends liberally from September to May^26^ and therefore the summer months of June, July, and August were excluded from our analyses. Daily-, zip code-specific concentrations of PM_2.5_^31^ represent fine particulate matter from all sources, including ambient levels and wildfire smoke. Daily hospital admissions for respiratory diseases (*n* = 1,655,011), which include pulmonary diagnoses such as asthma, chronic obstructive pulmonary disease (COPD), pneumonia, and interstitial lung disease were aggregated at the daily level by zip code.

**Fig. 2:**
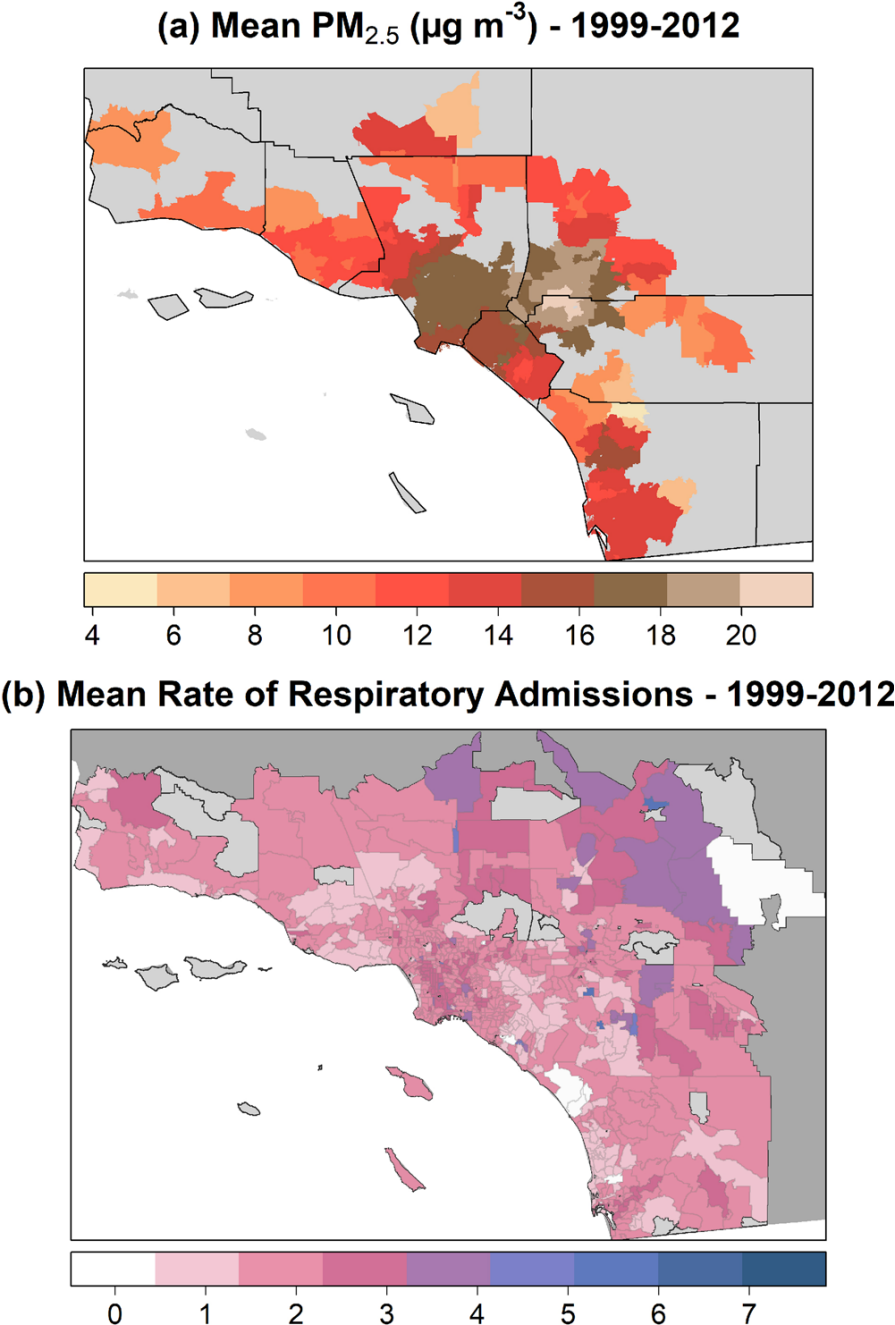
Mean values of PM_2.5_ and rate of respiratory admissions by ZIP code. **a** Mean PM_2.5_ concentrations at available zip codes (county boundaries shown in black) and **b** mean rate of respiratory admissions (per 100,000 individuals) per zip code during 1999–2012 (summer months not considered).

**Fig. 3:**
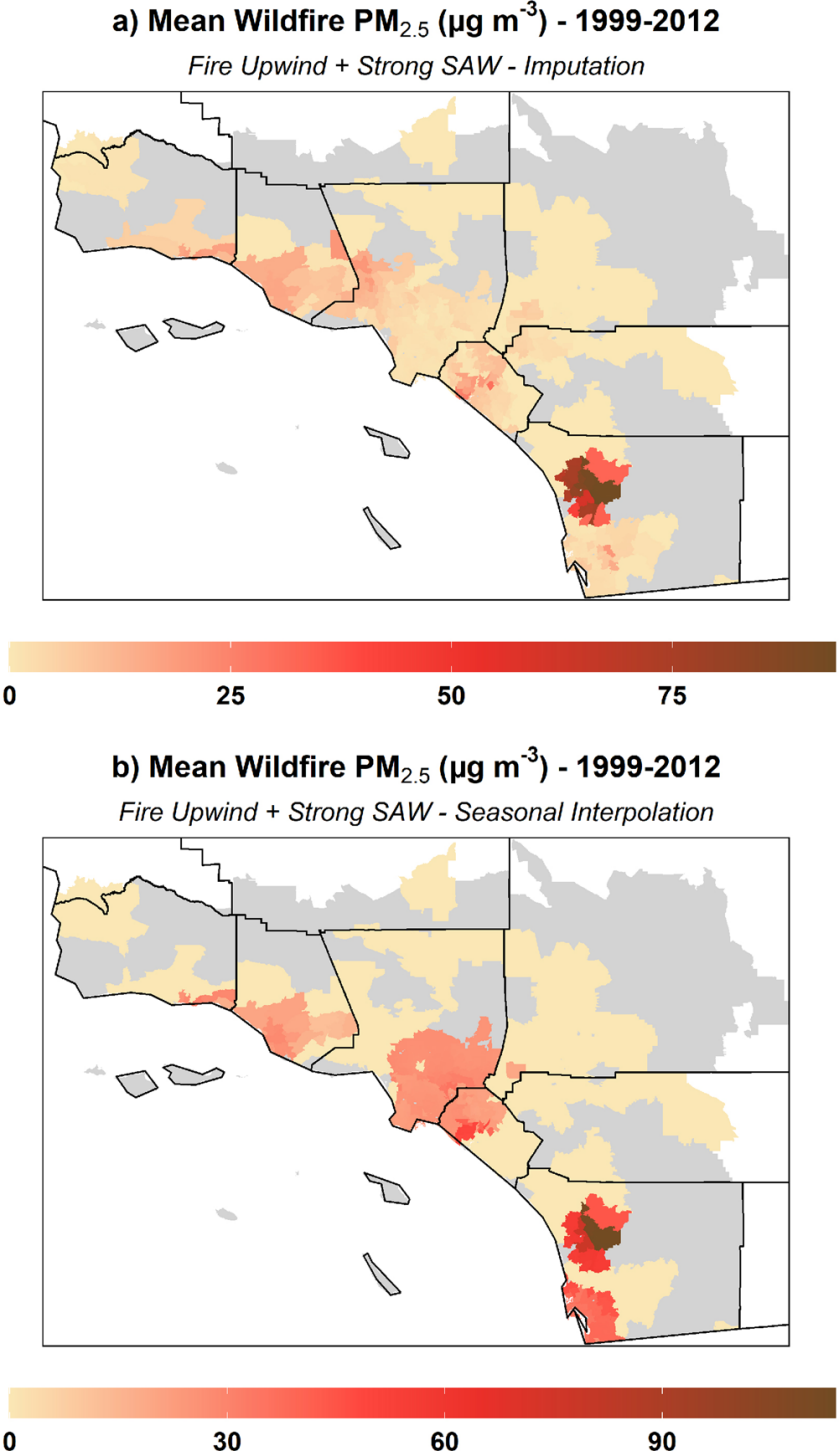
Wildfire-specific concentrations of PM_2.5_. Mean wildfire-specific PM_2.5_ estimated by **a** imputation and **b** seasonal interpolation methods and using the fire upwind and strong SAW exposure definition.

The highest mean PM_2.5_ concentrations were observed in highly populated coastal zip codes_,_ as well as in some inland zip codes in San Bernardino and Riverside Counties (Fig. 2a). Mean values for rates of respiratory admissions per 100,000 individuals suggested pockets of higher incidence in some urban areas and possibly heightened admissions in the Central Valley where dust may be a factor (Fig. 2b). Total monthly regional SAW activity, i.e., the sum of the Santa Ana Wind Regional Index (SAWRI)^26^ over a month within 1999–2012, reflecting both intensity and frequency of SAW, peaked between the months of November and January. Similarly, mean PM_2.5_ values were highest during late fall and winter months, whereas peaks in respiratory admissions were observed mainly in winter and particularly during February (Fig. S1 in Supplementary information). Figure 3 shows mean values for wildfire-specific PM_2.5_ estimated by imputation and seasonal interpolation, using the fire upwind and strong SAW exposure definition. In terms of the approaches used to isolate wildfire-specific PM_2.5_, when comparing the imputation and seasonal interpolation estimates, we find that the latter might yield larger values in some instances since the non-smoke background considers a seasonal median and thus can be lower than the non-smoke concentration imputed daily at a given zip code by means of the spatio-temporal imputation approach. In addition, we include case studies related to wildfire events that took place in October 2007, the most impactful in terms of wildfire smoke exposure and burden to public health^36^ (see details and resulting figure in Supplementary information). This case studies further illustrate that wildfire-specific PM_2.5_ estimates widely agree during extreme wildfire events such as the aforementioned 2007 firestorm.

## Results and discussion

### Effects of wildfire-PM_2.5_ on respiratory admissions

Table 1 summarizes our results for the effects of wildfire-PM_2.5_ on respiratory admissions in Southern California over the period 1999–2012, excluding summer months (June, July, and August), using the occurrence of strong SAWs and the presence of fire upwind as exposure definition. Based on the mean number (1.85) of daily respiratory admissions per 100,000 individuals, a 10 μg m^−3^ increase in PM_2.5_ was estimated to increase the number of admissions by only 0.76% (95% CI: 0.42–1.1). In contrast, the causal effects of PM_2.5_ attributable to wildfire smoke estimated by spatio-temporal imputation amounted to a 10.0% (95% CI: 3.5–16.5) increase in admissions, the highest percentage among all methods and exposures used. Such results were similar, though varying in the amplitude of % increase in admissions when using other approaches to isolate the wildfire-specific PM_2.5_ (see Table 1), as well as among all approaches considering smoke plumes within a 160 km buffer to define zip code days exposed to wildfire smoke (see Table S1 in Supplementary information). We conclude that wildfire-specific PM_2.5_ is up to 10 times more harmful on human health than PM_2.5_ from other sources. All the above methods and resulting estimates in increased admissions have, however, very wide confidence intervals, but these estimates are consistently higher than their aggregated or non-smoker counterparts.

**Table 1:** Effect of (wildfire and non-wildfire) PM_2.5_ on respiratory hospital admissions.

Fire upwind + strong SAW (1999–2012)	Regression model for respiratory admissions (rate per 100,000 people)							
	Aggregated sources (smoke and non-smoke)	Approach used to isolate wildfire-specific PM_2.5_						
		Instrumental Variable	Imputation		Interaction		Seasonal Interpolation	
		Wildfire-specific	Non-smoke	Wildfire-specific	Non-smoke	Wildfire-specific	Non-smoke	Wildfire-specific
PM_2.5_ coefficient	0.0014	0.0071	0.0013	0.018	1.00068	1.00061	0.0024	0.0055
(95% CI)	(0.00077–0.0021)	(−0.0022 to 0.017)	(0.00068–0.0020)	(0.0064–0.030)	(1.00049–1.00087)	(0.10–1.0015)	(0.0018–0.0030)	(−0.00068 to 0.012)
% change with 10 µg m^−3^ PM_2.5_	0.76	3.8	0.72	10	0.67	1.28	1.3	3.0
(95% CI)	(0.42–1.1)	(−1.2 to 8.9)	(0.36–1.1)	(3.5–16.5)	(0.48–0.86)	(0.37–2.19)	(0.97–1.7)	(−0.37 to 6.3)

All regressions include controls:flu admissions, weather covariates, day-of-week effects, month-of-year effects, zip code fixed effects, and a time trend.

Summer months (June, July, August) are excluded.

Mean PM_2.5_ = 15.6 μg m^−3^ (IQR = 9.2 μg m^−3^).

Mean rate of respiratory admissions per 100,000 people = 1.85.

Study limitations include the use of patient home address to estimate exposures and using community-level PM_2.5_ to assess and quantify individual wildfire PM_2.5_ exposures. The number and extent of smoke plumes used to categorize exposed zip code days represent a conservative estimate due to the limitations of visible satellite data. In addition to all the above, our definition of upwind fire exposure (detailed in “Methods”) may have also misclassified some of the smoke PM_2.5_ as non-smoke PM_2.5_ and vice versa. The fire upwind and strong SAW exposure definition focuses exclusively on SAW-driven wildfires and the overall north-easterly wind direction. The smoke plumes and buffer exposure definition, on the other hand, may include a few small non-SAW wildfires that occurred during the September–May period in a given year. Smoke from such inland wildfires, however, tends to be transported away from the coast by the prevailing onshore winds. This is also the case with summer wildfires. Although wildfires are burning in August 2020, at the time of this writing, Santa Ana winds are dormant in summer, and smoke from such wildfires does not typically impact the coastal zone. This is the reason summer was excluded from our analysis.

Additional potential limitations involve not including any lagged effects in our models when assessing the impact of exposure to smoke PM_2.5_ on respiratory health. Lastly, we acknowledge that wildfires can increase tropospheric ozone, which is a powerful oxidant that can irritate the airways and can thus increase the risk of hospitalizations for respiratory conditions^37^. At the same time, it has been shown that wildfires generate increases in ozone levels through processes distinct from PM_2.5_ from smoke^38,39^. Furthermore, a recent paper showed that PM_2.5_ was associated with respiratory ED visits and hospitalizations during a wildfire period even when adjusted for ozone^40^. Recent studies proposed different methods to predict ozone exposure during wildfire events^41^ and future studies should address isolating the health impacts of ozone generated specifically by wildfires.

### Implications for public health and air quality policy

Our findings indicate that wildfire-specific PM_2.5_ can cause a greater impact on respiratory health than PM_2.5_ from other sources. In each approach and each combination of variables to define zip code days exposed to wildfire plumes, we found that wildfire-specific PM_2.5_ were up to 10 times more harmful than non-smoke PM_2.5_. Wildfires have the potential to greatly and suddenly increase PM_2.5_ concentrations^22,36^, often surpassing safe limits (35 μg m^−^^3^) and reaching levels qualified as hazardous (>250 μg m^−3^) by the Air Quality Index (AQI, US EPA). Such sudden increase in PM_2.5_ caused by wildfire smoke can thus particularly affect vulnerable populations such as children and the elderly^23,36,42,43^. Overall, a greater impact of wildfire smoke PM_2.5_ on public health relative to ambient levels can be expected as PM_2.5_ concentration tends to be higher during wildfire episodes. However, in this study, we also show that even for similar exposure levels, PM_2.5_ from wildfires is considerably more dangerous for respiratory health. A comparable study^25^ examining the elderly population in counties across the Western US found a 7.2% increase in the risk of respiratory admissions during smoke days with high wildfire-specific PM_2.5_ (>37 μg/m) compared with nonsmoker days.

Recent toxicological studies have shown differences in the composition and effects of wildfire PM_2.5_ compared to ambient sources^14,15,44,45^. In one study^14^, significant changes were observed in macrophage and neutrophil counts in mouse lung samples exposed to wildfire particulate matter compared to ambient sources. Specifically, the authors observed that the toxicity of PM in wildfire smoke to the respiratory system is 3–4 times greater than equivalent doses of ambient PM^14^. A subsequent study by Wegesser et al.^44^. expanded on these findings to show that substances such as polycyclic aromatic hydrocarbons can be present in much higher concentrations in smoke versus levels detected in ambient air. Another study^45^ examined the inflammatory responses due to wildfire smoke PM exposure and found significant changes in reactive oxygen species and subsequent oxidative stress, leading to higher cell degeneration and potential programmed cell death.

In addition to differences in the chemical composition of smoke and ambient PM, different stages of biomass combustion appear to have differential impacts on health^15^. Recent findings have suggested that the types of trees and the temperature at which the combustion takes place may explain the differential toxicity regarding wildfire-specific PM, as observed in mouse lung response^15^. All the above evidence suggests that the assumption that all particles of a given size class have the same toxicity (which is currently the basis for regulation of airborne PM_2.5_) may be inaccurate. Future studies should address the epidemiological response to wildfires affecting different ecosystems and fuel types and burning at different combustion temperatures.

Understanding the impacts of wildfire on public health is of vital importance in Southern California where several factors may increase exposure to wildfire-specific PM_2.5_ in the context of global climate change. Wildfire severity and risk in this region will likely intensify in the warming future^46^ as changing precipitation and wind patterns gradually push the wildfire season from fall to winter when back-to-back SAWs can cause wildfires to burn longer^29,47,48^. In addition, given that most large fires in Southern California are caused by human ignitions, whether accidental or deliberate, the current and projected population growth trends and the expansion of the Wildland-Urban Interface may create additional wildfire ignitions^49^ in the region. Our results could be transferred to similar regions in the US and the world where wind-driven wildfires cause damage to public health (via smoke PM_2.5_) and property, particularly in a changing world scenario where wildfire-PM_2.5_ is projected to increase relative to emissions from other sources.

## Methods

The following sections describe the data and methodology used to estimate wildfire-specific PM_2.5_ and to quantify its impact on respiratory health. We used ArcGIS 10.5^50^, R version 3.5.1^51^, and Stata version 16^52^ for all analyses detailed below.

### Respiratory health

Daily hospital admissions for respiratory diseases were obtained from the California Office of Statewide Health Planning and Development (OSHPD) database of patient discharge data for the study period and spatial domain. Respiratory hospitalizations correspond to the ICD 9 codes 460:519 which include pulmonary diagnoses, such as asthma, COPD, pneumonia, and interstitial lung disease. In addition, data for flu diagnosis were also available. All data were aggregated at the daily level by zip code and converted to rates of admission by dividing the admission counts by the population.

### Fine particulate matter (PM_2.5_)

Daily-, zip code-specific concentrations of PM_2.5_ were estimated from 1999 through 2012^31^ using 24-h daily means sampled and analyzed by the US EPA Air Quality System (https://www.epa.gov/aqs) at ground monitoring stations within a 20 km radius of each population-weighted zip code centroid. Values were interpolated using an inverse distance weighting approach^31^, which gives greater importance to monitoring stations closer to the point of interest. These PM_2.5_ values, coming from monitoring station data, represent fine particulate matter from all sources, including ambient levels and wildfire smoke. PM_2.5_ values were available on 578 zip codes in our study region, subject to monitoring station data availability, with varying degrees of missing data. Overall, the mean percentage of missing values in these zip codes was 33% (median: 15%).

### Wildfire upwind and strong Santa ana winds

**Fig. 4:**
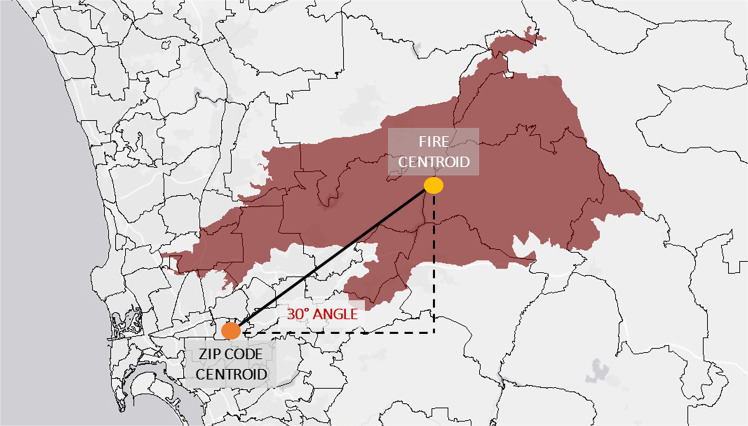
Wildfire upwind exposure estimation. The relationship between a given zip code centroid and a fire centroid is assessed by means of geospatial tools. Fires burning upwind are considered within the context of the north-easterly direction of Santa Ana Winds. We use the distance between centroids, as well as the angle of the spatial relationship as shown here, to classify a zipcode day as exposed (or not).

Wildfire exposure was derived using fire perimeters in Southern California from 1999 to 2012 from the Fire and Resource Assessment Program of the California Department of Forestry and Fire Protection (CalFire; http://frap.fire.ca.gov/). Zipcodes were assigned a wildfire event upwind if their centroids were within a 160-km radius of the fire perimeter’s centroid (Fig. 4) and were located within a range of angles (roughly from −10° to 120°) that capture the north-easterly direction and the smoke plumes associated with Santa Ana winds. Neighboring zip codes to the fire perimeter were also assigned a wildfire event. Wildfire conditions were defined as the days during which a wildfire was detected upwind of a given zip code.

The combination of strong SAWs and wildfire upwind is the condition under which smoke is expected to impact communities downwind of a wildfire. We used the daily version of the original hourly SAWRI^26^, evaluated over the Santa Ana wind domain, to identify days with strong and widespread SAWs. SAWRI, expressed in m s^−1^, provides an observationally validated regional daily summary of the dynamically downscaled SAWs. Strong Santa Ana wind conditions were defined as any Santa Ana wind day when SAWRI was above 3.06 m s^−1^ (its median value, conditional on taking a positive value).

### Smoke plumes

Smoke plumes were obtained from the NOAA Hazard Mapping System (HMS), available from September 2005 onward. The HMS product uses visible satellite imagery and trained satellite analyst skills to estimate the spatial extent of smoke, though it cannot discern whether a given plume is at ground level or higher in the atmosphere^53^. In addition, the HMS smoke-plume extent data has not been validated and could thus have systematic biases because discrimination of smoke can vary by region, season, and weather conditions^54^. However, HMS smoke plumes remain a common binary metric used to determine if smoke is present in the atmospheric column on a given day^35^. The HMS smoke products are stored as polygon shapefiles representing the spatial extent of daily smoke plumes (ftp://satepsanone.nesdis.noaa.gov/volcano/FIRE/HMS_ARCHIVE/). A simple smoke binary variable was created by intersecting zip code polygons with smoke polygons, which was then used as an indication of daily exposure to wildfire PM_2.5_. We included the additional condition of wildfire presence within a 160-km radius from a given zip code in order to classify it as exposed to wildfire smoke.

### Weather covariates

We collected hourly data from NOAA’s National Centers for Environmental Information Integrated Surface Database (NCEI ISD; https://www.ncdc.noaa.gov/isd) and calculated 24-h daily means for wind speed, temperature, and humidity. Values were interpolated using an inverse distance weighting approach^31^ and considering the daily observations from monitoring stations within a 20 km radius of each population-weighted zip code centroid.

### Estimating wildfire-specific PM_2.5_

#### Instrumental variable (IV) approach: a two-stage regression

We use a novel joint instrument within a two-stage regression based on both wind and wildfire occurrence. Specifically, we modeled the incidence of wildfire upwind during strong Santa Ana winds, in order to isolate the effects of wildfire-specific PM_2.5_ on respiratory admissions. Indeed, through using these joint instruments and a two-stage least square approach, we estimate the local average treatment effect of PM_2.5_ on respiratory hospital admissions^55^. Said differently, this procedure allows us to isolate “complier PM_2.5_ values” regarding the presence an upwind wildfire smoke in a given zip code-day. In this context, monotonicity implies that the joint instruments have no effect on PM_2.5_ levels on non-wildfire zip code-days and that all zip code-days with upwind wildfire are affected in the same way. Furthermore, using this approach we can assume that any effect the joint IV has on the hospital admissions is only mediated by a “local” variation of PM_2.5_ for eligible zip code-days only (exclusion restriction criteria) while having a strong correlation between the joint IV and PM_2.5_ levels.

In the first stage (Eq. 1, below), PM_2.5_ is regressed on a binary variable combining the presence of wildfire upwind and strong SAWs. In the second stage (Eq. 2), rates of respiratory admissions per 100,000 individuals are regressed on the fitted values of the explanatory variables, including wildfire PM_2.5_ estimated in the previous stage. For the IV estimation to be consistent, all exogenous variables used in the second stage must also be included in the first stage and only one exogenous variable coefficient may be estimated in the second stage for each instrumental variable included in the first stage regression^56^. The following controls were included: the daily number of flu admissions by zip code, weather covariates: mean daily wind speed, temperature and humidity, day-of-week effects, month-of-year effects, a linear time trend, and zip code fixed effects. Ordinary least squares regressions were implemented with the plm R-package for panel data^57^.

First stage regression

1 $${\mathrm{PM}}_{2.5\,it} 	= \gamma _0 + \gamma _1{\mathrm{Exposure}}\;{\mathrm{Definition}}_{it} + \gamma _2{\mathrm{Flu}}_{it} + \gamma _3{\mathrm{Wind}}_{it} + \gamma _4{\mathrm{Temperature}}_{it} \\ 	\quad+\, \gamma _5{\mathrm{Humidity}}_{it} + {\mathrm{Zip}}_i + {\mathrm{Weekday}}_t + {\mathrm{Month}}_t + \tau t + u_{it}$$

Second stage regression

2 $${\mathrm{Resp}}_{it} 	= \beta _0 + \beta _1\widehat {PM_{2.5}}_{it} + \beta _2{\mathrm{Flu}}_{it} + \beta _3{\mathrm{Wind}}_{it} + \beta _4{\mathrm{Temperature}}_{it} + \beta _5{\mathrm{Humidity}}_{it}\\ 	\quad + {\mathrm{Zip}}_i + {\mathrm{Weekday}}_t + {\mathrm{Month}}_t + \tau t + {\it{ \in }}_{it}$$

### Spatio-temporal multiple imputation approach

For this approach, we used a cubic spline interpolation to impute the PM_2.5_ concentrations attributable to non-smoke sources in zip code/days identified as exposed to wildfire smoke. Cubic splines are an extension of polynomial regression where times *t* are divided into *k* intervals called knots. For each interval, a regression is fit with three parameters. This method has been found to allow the inclusion of local characteristics of a trend without prejudicing its global characteristics.

More specifically, we followed the steps below:

- Using the exposure definition of wildfire upwind and strong SAWs (or smoke plumes; Sections 3 and 4), we identified the zip code days exposed to wildfire smoke in our original PM_2.5_ dataset.
- We used a spline interpolation approach to impute the values of non-smoke PM_2.5_ on all zip code days categorized as exposed to smoke and where PM_2.5_ data were originally available (i.e., we did not impute missing values in the original dataset). Cubic spline interpolation was implemented in R by means of the imputeTS package^58^. This step provided estimates of ambient PM_2.5_ unrelated to wildfire smoke.
- We then subtracted all non-smoke PM_2.5_ values from the original daily PM_2.5_ concentrations to obtain the levels of PM_2.5_ attributable to wildfire smoke in zip code days previously categorized as exposed.
- Lastly, rates of respiratory admissions are regressed on the wildfire-specific PM_2.5_ concentrations (Eq. 3), including controls for the daily number of flu admissions by zip code, day-of-week effects, month-of-year effects, a linear time trend, and zip code fixed effects.
  3 $${\mathrm{Resp}}_{it} =	 \; \beta _0 + \beta _1{\mathrm{Wildfire - PM}}_{2.5\,it} + \beta _2{\mathrm{Flu}}_{it} + \beta _3{\mathrm{Wind}}_{it} + \beta _4{\mathrm{Temperature}}_{it} \\ 	 +\beta _5{\mathrm{Humidity}}_{it} + {\mathrm{Zip}}_i + {\mathrm{Weekday}}_t + {\mathrm{Month}}_t + \tau t + {\it{ \in }}_{it}$$

### Interaction model

We used a zip code fixed effects Poisson regression model to quantify the effects of wildfire-specific and non-wildfire-specific PM_2.5_ on respiratory admissions. The rate of admissions was modeled as a function of the interaction between wildfire exposure and PM_2.5_ and a set of control variables comprised of the number of flu admissions, mean daily wind speed, mean daily temperature, mean daily humidity, zip code fixed effects, dummy variables for day of week and month of the year, a linear time trend, and log-transformed zip code-specific population as an offset term (Eq. 4).

4 $${\mathrm{Resp}}_{it} =	 \exp ( \log ( {{\mathrm{Population}}_{it}} ) + \beta _1{\mathrm{PM}}2.5_{it} + \beta _2{\mathrm{Wildfire}}_{it} + \beta _3{\mathrm{PM}}2.5_{it} \\ 	 \times {\mathrm{Wildfire}}_{it} + \beta _4{\mathrm{Flu}}_{it} + \beta _5{\mathrm{Wind}}_{it} + \beta _6{\mathrm{Temperature}}_{it} + \beta _7{\mathrm{Humidity}}_{it} \\ 	 + {\Gamma}_1{\mathrm{Zip}}_i + {\Gamma}_2{\mathrm{Weekday}}_t + {\Gamma}_3{\mathrm{Month}}_t + \tau \,t + {\it{ \in }}_{it} )$$

Using this framework, the effect of non-wildfire PM_2.5_ is captured by β1, and the effect of wildfire PM_2.5_ is captured by the sum of β1 and β3, which are then transformed to obtain the marginal effects of changes in non-wildfire-specific and wildfire-specific PM_2.5_. Standard errors and confidence intervals on the marginal effects are obtained using the delta method.

### Seasonal interpolation

We based this approach on the method implemented by Lipner et al.^27^ to segregate wildfire smoke PM_2.5_ from other sources of PM_2.5_:

- As in the imputation approach, we identified the zip code days exposed to wildfire smoke in our original PM_2.5_ dataset.
- Subsequently, daily non-smoke PM_2.5_ was provisionally estimated by means of inverse distance weighting spatial interpolation considering PM_2.5_ data from only zip code days not exposed to wildfire smoke.
- Seasonal non-smoke PM_2.5_ was then calculated as the median of the non-smoke PM_2.5_ estimates above, for each three-month season and grid cell.
- These seasonal non-smoke PM_2.5_ concentrations were subtracted from the original PM_2.5_ dataset (setting negative differences to zero) to compute PM_2.5_ derived from wildfire smoke. The remaining component of daily PM_2.5_ (equal to the seasonal background unless the background was higher than the full daily PM_2.5_ concentration) was then defined to be the zip code-specific, daily non-smoke PM_2.5_ concentration.
- Rates of respiratory admissions are regressed on the wildfire-specific PM_2.5_ concentrations (Eq. 5), including controls for the daily number of flu admissions by zip code, day-of-week effects, month-of-year effects, a linear time trend, and zip code fixed effects.
  5 $${\mathrm{Resp}}_{it} =	 \; \beta _0 + \beta _1{\mathrm{Wildfire - PM}}_{2.5\,it} + \beta _2{\mathrm{Flu}}_{it} + {\mathrm{Zip}}_i \\ 	 + {\mathrm{Weekday}}_t + {\mathrm{Month}}_t + \tau \,t + {\it{ \in }}_{it}$$

### Reporting summary

Further information on research design is available in the Nature Research Reporting Summary linked to this article.

## Supplementary information

Supplementary Information

Reporting Summary

## Data Availability

The data, with the exception of health data, that support the findings of this study are available from the corresponding author upon reasonable request. Publicly available data is also found here: daily mean PM_2.5_ concentrations at EPA monitoring sites: https://www.epa.gov/aqs; CalFire Wildfire Perimeters: http://frap.fire.ca.gov; NOAA Hazard Mapping System smoke plumes: ftp://satepsanone.nesdis.noaa.gov/volcano/FIRE/HMS_ARCHIVE/; meteorological variables from NOAA’s National Centers for Environmental Information Integrated Surface Database: https://www.ncdc.noaa.gov/isd.

## Code availability

Scripts that support the findings of this study are available from the corresponding author upon reasonable request.
