# Supplementary material for: Wildfire smoke impacts respiratory health more than fine particles from other sources: observational evidence from Southern California
Source: Nat Commun. 2021 Mar 5;12:1493. doi: 10.1038/s41467-021-21708-0 (PMC7935892; doi:10.1038/s41467-021-21708-0)
Supplement: Supplementary file 1 — Supplementary Information [file 41467_2021_21708_MOESM1_ESM.pdf]

Supplemental Information

Wildfire smoke impacts respiratory health more than fine particles from other sources: observational evidence from Southern California

Rosana Aguilera [1§*], Thomas Corringham [1§], Alexander Gershunov [1], Tarik Benmarhnia [1,2]

[1] Scripps Institution of Oceanography, University of California San Diego, La Jolla, CA, USA
[2] Herbert Wertheim School of Public Health and Human Longevity Science, University of California San Diego, La Jolla, CA, USA

§ These authors contributed equally

*Address correspondence to:
R. Aguilera, Scripps Institution of Oceanography, 9500 Gilman Drive #0230, La Jolla, CA, 92093, USA. Telephone: (805) 280-6219, E-mail: r1aguilerabecker@ucsd.edu

Table S1: Effect of (Wildfire and non-Wildfire) $PM_{2.5}$ on Respiratory Hospital Admissions

	Regression Model for Respiratory Admissions (rate per 100,000 people)							
Smoke Plumes and Fire Buffer (2005-2012)	Aggregated sources (smoke and non-smoke)	*Approach used to isolate wildfire-specific $PM_{2.5}$*						
		IV	Imputation		Interaction		Seasonal Interpolation	
		Wildfire-specific	Non-smoke	Wildfire-specific	Non-smoke	Wildfire-specific	Non-smoke	Wildfire-specific
$PM_{2.5}$ (95% CI)	0.000776 (-0.00035, 0.0019)	0.00213 (0.000012, 0.00426)	0.00061 (-0.0095- 0.0019)	0.015991 (0.00585- 0.0261)	1.0010 (1.00071, 1.0014)	1.0056 (1.0030, 1.0082)	0.00025 (-0.00076, 0.0013)	0.0045 (0.0053, 0.021)
% change with 10 ug m^{-3} $PM_{2.5}$ (95% CI)	0.4 (-0.2, 1.0)	1.2 (0.0, 2.3)	0.3 (-5.2, 5.8)	8.6 (3.2, 4.1)	1.04 (0.71, 1.37)	6.65 (4.08, 9.23)	0.13 (-0.41, 0.67)	8.1 (3.3, 13.0)

All regressions include controls:

flu admissions, weather covariates, day-of-week effects, month-of-year effects, zip code fixed effects, and a time trend

Summer months (June, July, August) are excluded

Mean $PM_{2.5}$ = 15.6 μg m^{-3} (IQR = 9.2 μg m^{-3})

Mean rate of respiratory admissions per 100,000 people = 1.85

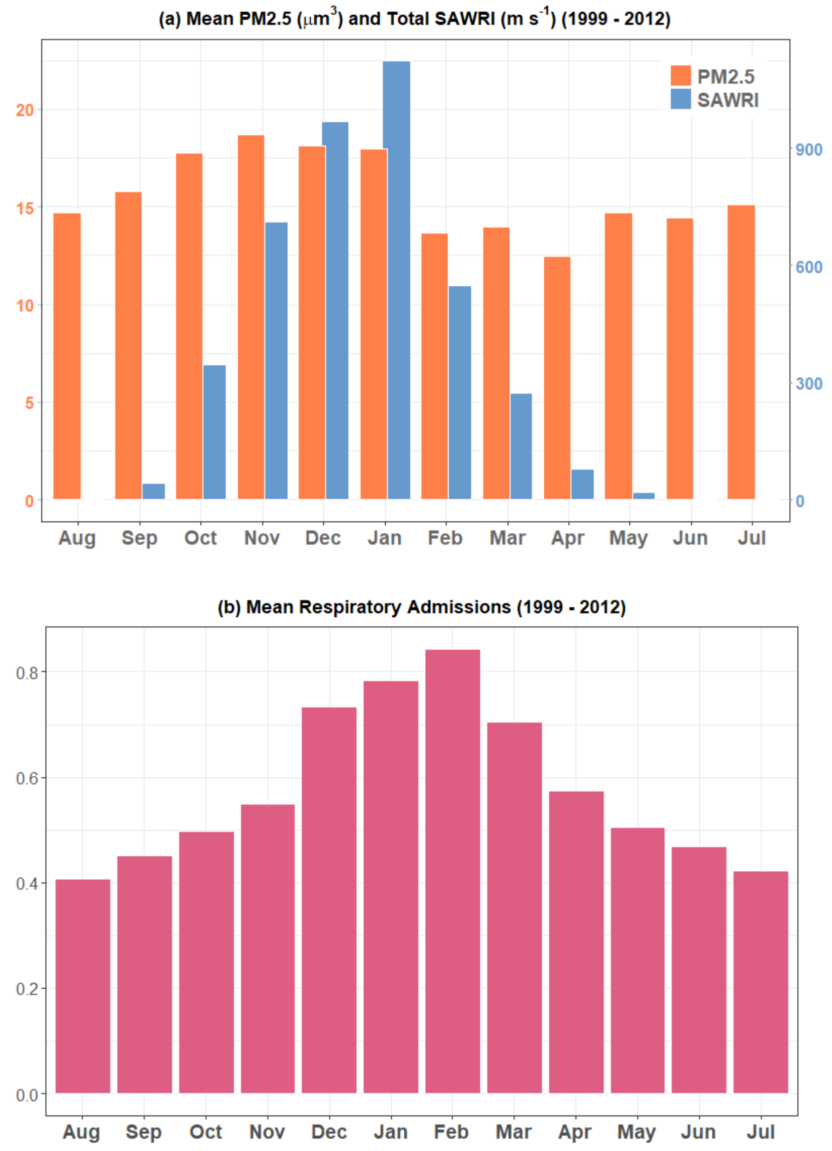

Figure S1: Seasonality of (a) mean $PM_{2.5}$ levels and SAWRI total activity, and (b) mean respiratory admissions during 1999-2012.

Supplementary Note 1: Spatial variation of wildfire-specific $PM_{2.5}$

To illustrate the spatial variation of our estimates for wildfire-specific $PM_{2.5}$, we focus on a set of wildfire events that took place in October 2007, the most impactful in terms of wildfire smoke exposure and burden to public health (e.g., Hutchinson et al., 2018) within the common period of the exposure definitions used (2005-2012).

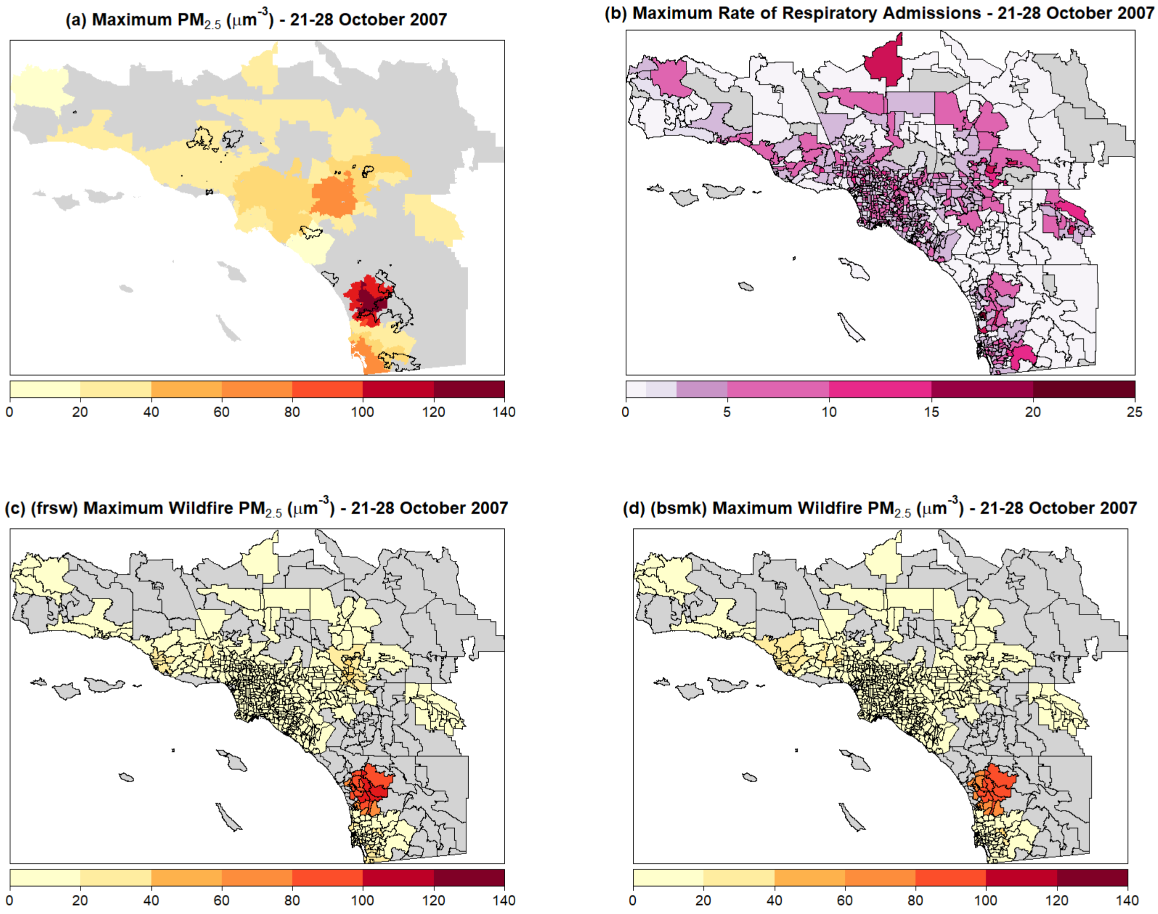

Figure S2: Wildfire perimeters are shown in black in (a). Maximum levels during the week of October 21-28, 2007 for (a) $PM_{2.5}$, (b) rate of respiratory admissions per 100,000 individuals and wildfire-specific $PM_{2.5}$ estimated via imputation using the smoke exposure definitions of strong SAW-driven wildfires (frsw; c) and smoke plumes and a 100mi buffer from the fire perimeter (bsmk; d).

Supplementary Note 2: Sensitivity analysis related to missing data

To test the effect of missing values in the 578 zip codes involved in our study, we fitted univariate models for each zip code using a fast missing value imputation method by chained random forest and used the resulting $PM_{2.5}$ data (i.e., without any data gaps) in our four approaches with each of the two exposures to assess the impact of wildfires on health (Table S2, below). In some instances, the imputed values slightly improved our estimation but most importantly, wildfire-specific $PM_{2.5}$ was consistently more harmful than $PM_{2.5}$ from other sources (though, again, confidence intervals were wide and some estimates were not significant).

Table S2: Effect of Imputed $PM_{2.5}$ on Respiratory Hospital Admissions

		Fire Upwind + Strong SAW (1999-2012)						
		Approach used to isolate wildfire-specific $PM_{2.5}$						
	Aggregated sources (smoke and non-smoke)	IV	Imputation		Interaction		Seasonal Interpolation	
		Wildfire-specific	Non-smoke	Wildfire-specific	Non-smoke	Wildfire-specific	Non-smoke	Wildfire-specific
$PM_{2.5}$ (95% CI)	0.00199 (0.00147, 0.0025)	0.0050 (0.0035, 0.0064)	0.00192 (0.00141, 0.0024)	0.0285 (0.0191, 0.038)	1.000737 (1.000564, 1.000911)	0.9998 (0.9990, 1.00067)	0.0023 (0.00166, 0.00296)	0.00754 (0.000899, 0.0142)
% change with 10 ug m^{-3} $PM_{2.5}$ (95% CI)	1.07 (0.79, 1.35)	2.7 (1.9, 3.5)	1.04 (0.76, 1.32)	15.4 (10.3, 20.5)	0.74 (0.56, 0.91)	0.54 (-0.3, 1.39)	1.25 (0.90, 1.6)	4.08 (0.49, 7.7)
		Smoke Plumes and Fire Buffer (2005-2012)						
		Approach used to isolate wildfire-specific $PM_{2.5}$						
	Aggregated sources (smoke and non-smoke)	IV	Imputation		Interaction		Seasonal Interpolation	
		Wildfire-specific	Non-smoke	Wildfire-specific	Non-smoke	Wildfire-specific	Non-smoke	Wildfire-specific
$PM_{2.5}$ (95% CI)	0.00082 (-0.00014, 0.0018)	0.0024 (-0.00055, 0.0053)	0.00082 (-0.00014, 0.0018)	0.0039 (-0.0072, 0.015)	1.000169 (0.9999151, 1.000422)	1.004601 (1.002354, 1.006852)	0.00037 (-0.00081, 0.0015)	0.013 (0.0062, 0.021)
% change with 10 ug m^{-3} $PM_{2.5}$ (95% CI)	0.44 (-0.076, 0.96)	1.3 (-0.3, 2.8)	0.45 (-0.073, 0.96)	2.1 (-3.9, 8.1)	0.17 (-0.08, 0.42)	4.75 (2.53, 6.98)	0.20 (-0.43, 0.83)	7.3 (3.3, 11.3)
